# Limited resection is comparable to lobectomy for tumor size ≤ 2 cm pulmonary invasive mucinous adenocarcinoma

**DOI:** 10.1186/s12957-024-03387-5

**Published:** 2024-04-25

**Authors:** Weikang Lin, Hang Su, Huikang Xie, Long Xu, Tingting Wang, Long Wang, Xuefei Hu, Deping Zhao, Yuming Zhu, Haifeng Wang, Gening Jiang, Dong Xie, Chang Chen, on behalf of the Surgical Thoracic Alliance of Rising Star Group

**Affiliations:** 1 Department of Thoracic Surgery, Shanghai Pulmonary Hospital, Tongji University School of Medicine, Shanghai, 200433 People’s Republic of China; 2 Clinical Center for Thoracic Surgery Research, Tongji University, Shanghai, People’s Republic of China; 3 Department of Pathology, Shanghai Pulmonary Hospital, Tongji University School of Medicine, Shanghai, People’s Republic of China

**Keywords:** Limited resection, Lobectomy, Invasive mucinous adenocarcinomas

## Abstract

**Objectives:**

Invasive mucinous adenocarcinoma (IMA) has a rare incidence with better prognosis than nonmucinous adenocarcinoma. We aimed to investigate the prognosis between limited resection and lobectomy for patients with clinical stage IA IMA ≤ 2 cm.

**Methods:**

Data were taken from two cohorts: In Shanghai Pulmonary Hospital (SPH) corhort, we identified 403 patients with clinical stage IA IMA who underwent surgery. In the SEER corhort, 480 patients with stage T1 IMA who after surgery were included. Recurrence-free survival (RFS) for SPH corhort, lung cancer–specific survival (LCSS) for the SEER corhort and overall survival (OS) for both corhort were compared between patients undergoing lobectomy and limited resection by Log-rank and Cox proportional hazard regression model.

**Results:**

In SPH corhort, patients who underwent limited resection had equivalent prognosis than those underwent lobectomy (5-year RFS: 79.3% *versus*. 82.6%, *p* = 0.116; 5-year OS: 86.2% *versus.* 88.3%, *p* = 0.235). However, patients with IMA > 2 to 3 cm had worse prognosis than those with IMA ≤ 2 cm (5-year RFS: 73.7% versus. 86.1%, *p* = 0.007). In the analysis of IMA > 2 to 3 cm subgroup, multivariate analysis showed that limited resection was an independent risk factor of RFS (hazard ratio, 2.417; 95% confidence interval, 1.157–5.049; *p* = 0.019), while OS (*p* = 0.122) was not significantly different between two groups. For IMA ≤ 2 cm, limited resection was not a risk factor of RFS (*p* = 0. 953) and OS (*p* = 0.552). In the SEER corhort, IMA ≤ 2 cm subgroup, limited resection was equivalent prognosis in LCSS (*p* = 0.703) and OS (*p* = 0.830).

**Conclusions:**

Limited resection could be a potential surgical option which comparable to lobectomy in patients with clinical stage IA IMA ≤ 2 cm.

**Supplementary Information:**

The online version contains supplementary material available at 10.1186/s12957-024-03387-5.

## Background

In 2015, the World Health Organization (WHO) classification of lung cancers, considered invasive mucinous adenocarcinoma (IMA) as a variant subtype of invasive adenocarcinoma (ADC) [1]. The distinctive histological morphology is characterized by goblet or columnar cells with abundant intracytoplasmic mucin. Compared to nonmucinous ADC, IMA accounts for nearly 5% of all kinds of lung ADCs and had striking differences in its clinical, radiological, pathological, and genetic aspects [2, 3]. More importantly, patients with IMA have a more favorable prognosis than those with nonmucinous ADC. Furthermore, previous studies have reported that patients with IMA have a comparable prognosis to patients with acinar and papillary subtypes nonmucinous ADC [4, 5].

With the increasing detection rate of early-stage lung cancer using thin-section computed tomography, limited resection aroused great interest as a therapy for early-stage lung cancers [6, 7]. Despite ongoing controversies about the oncologic efficiency between limited resection and lobectomy, its use in early-stage lung cancers is increasing [8]. In clinical practice, limited resection is generally considered acceptable for early-stage lung ADC presenting as ground glass opacity-dominant (GGO) nodules [9, 10]. However, there is little evidence to support the appropriateness of limited resection as a treatment for patients with early-stage IMA.

Hence, we used the patients data from Shanghai Pulmonary Hospital and the Surveillance, Epidemiology, and End Results (SEER) database to investigate the potential candidates with clinical stage IA IMA who were benefited from limited resection.

## Patients and methods

### Patient selection

*Shanghai Pulmonary Hospital.* This retrospective study was approved by the Institutional Review Board of Shanghai Pulmonary Hospital. Data were obtained from patients with clinical stage IA IMA who underwent surgical treatment in Shanghai Pulmonary Hospital from January 2012 to December 2017. Inclusion criteria were as follows: (1) Tumor size ≤ 3 cm on computed tomography (CT) scans; (2) tumors that were pathologically diagnosed as pure IMA or mixed mucinous and nonmucinous ADC; and (3) aged between 20 and 80 years old. Patients with mucinous adenocarcinoma in situ or minimally invasive mucinous adenocarcinoma were excluded. Patients with multiple nodules or had the concomitant presence of other malignancies were also excluded. We also excluded the patients who underwent preoperative chemotherapy or had history of malignant tumors. All the patients were staged according to the 8th TNM staging system [11]. The end date of follow-up was December 1, 2020.

*SEER database.* We used the SEER 18 Registry data including the Hurricane Katrina Impacted Louisiana Cases, Nov 2017 Sub (1973–2015 varying) for this analysis. This SEER database was queried for IMA of the lung from January 1, 2010 to December 31, 2015 based on the 3rd edition codes for IMA of the lung (8253, 8254, 8480). Patients were included when the following inclusion criteria were met: T1a or T1b (tumor size ≤ 2 cm or 2–3 cm) based on AJCC (American Joint Committee on Cancer) 7th ed from column of SEER database, lobectomy, limited resection and presence of one primary cancer only. The age older than 80 and younger than 20 were also excluded.

### Surgical treatment for the Shanghai pulmonary hospital cohort

In our hospital, patients with clinical stage IA lung mucinous ADC were more likely to have undergone lobectomy. However, limited resection was also performed after a comprehensive evaluation. There were mainly two indications of limited resection in our hospital. The main candidate group for limited resection was the patients deemed unfit or at high risks for a lobectomy, including underlying pulmonary disease and/or heart disease and advanced age, according to previous studies [12–15]. The second indication was that patients were required to meet all the following criteria according to previous studies: [16]: (i) ≤ 3 cm in maximum diameter with radiologically non-invasive appearance (consolidation/tumors ratio < 0.5), (ii) location within the outer third of the lung parenchyma.

For lobectomy, systemic lymph node dissection is mandatory, including hilar lymph node and at least three stations of mediastinal lymph node from 2R, 4R, 7, 8, 9 for the right side and 4 L, 5, 6, 7, 8, 9 for the left side, respectively. Limited resection was included wedge resection and segmentectomy. Most of the patients underwent systemic lymph node dissection for segmentectomy. However, if patient conditions do not permit, it is acceptable to remove mediastinal lymph nodes during segmentectomy. For wedge resection, selective mediastinal lymph node dissection was accepted and the selective sample of the mediastinal lymph node was also allowed after a comprehensive evaluation by the surgeons [17].

A resection margin of more than 2 cm was ensured following a comprehensive preoperative examination and discussion before chosing the surgical strategy. And the pathologic diagnosis would check the resection margin for subsequent treatment decisions.

### Radiological and pathological evaluation for the Shanghai pulmonary hospital cohort

All chest CT scans and tumor size were re-evaluated by two radiologists, respectively. When a disagreement occurred, a third senior radiologist was required for an accurate diagnosis. The size of the tumors was measured as the largest axial diameter of the lesion on the lung window setting [level, -500 Hounsfield unit (HU); width, 1350 HU].

All available hematoxylin and eosin-stained slides were reviewed by two pathologists independently in SPH cohort. If disagreement occurred, the third senior pathologist would join the discussion to reach a final consensus. The criteria for histological subtyping were performed according to the 2015 WHO lung ADC classification [1]. The definition of IMA was tumors having columnar or goblet cells with abundant intracellular mucin and with lepidic, acinar, papillary, micropapillary, or solid pattern. Each histologic pattern was recorded in 5% increments. According to the percentage of mucinous and nonmucinous components, IMAs were divided into two groups: pure IMA (with > 90% invasive mucinous pattern) and mixed invasive mucinous and nonmucinous ADCs (with ≥ 10% of each pattern of mucinous and nonmucinous). Tumor size, visceral pleural invasion (VPI), and lymph node involvement were recorded as pathologic features.

### Clinical follow-up and outcome for the Shanghai pulmonary hospital cohort

All the patients included in this study were regularly monitored for at least three years from the date of surgery, and the patients before December 1, 2016 were followed-up for more than five years. For the first two years, physical examination, chest CT and serum carcinoembryonic antigen (CEA) measurements were performed at least twice per year. Thereafter, physical examinationand chest CT scans would be performed semiannually for years three through five post-surgery. Additional examinations, including radionuclide bone scans and magnetic resonance imaging of the brain were performed when patients had any signs of recurrence or symptom occurred. Recurrence-free survival (RFS) and overall survival (OS) were calculated from the date of surgery to the date of first recurrence or death, respectively.

### Statistical analysis

All clinical data are either shown as mean ± standard deviation or number (percent values). The Pearson *χ*2 test was conducted to compare categorical variables, and an independent sample *t* test was used to compare continuous variables. Log-rank test and Cox proportional hazard regression model were applied to evaluate predictive factors for recurrence-free survival (RFS) for Shanghai Pulmonary Hospital corhort, lung cancer–specific survival (LCSS) for the SEER corhort and overall survival (OS) for both corhort. All the statistical analyses were performed in SPSS version 21.0 (IBM, Armonk, NY) and statistical significance was set as *p* < 0.05.

## Result

### The Shanghai pulmonary hospital cohort

#### Patient characteristics

A total of 403 patients with clinical stage IA IMA were met our inclusion criteria. Among them, 316 patients (78.4%, 316/403) underwent lobectomy, and 87 patients (21.6%, 87/403) were treated by limited resection. Among limited resection group, 28 patients underwent segmentectomy and 59 patients underwent wedge resection. Patients were classified into two groups according to surgical types and the clinicopathological characteristics were summarized and compared in Table 1. There are no significant differences between patients in limited resection group and lobectomy group in terms of age (*p* = 0.156), sex (*p* = 0.149), smoking status (*p* = 0.626), CEA (*p* = 0.432), %predicted forced expiratory volume in 1 s (FEV 1) (*p* = 0.103), cardiovascular disease *(p* = 0.083), diabetes mellitus (*p* = 0.103). Patients in the limited resection group had smaller tumor size than those in the lobectomy group. The median follow-up time was 46 months (range, 24 to 82 months) and 43 months (range, 21 to 80 months) in lobectomy and limited resection groups, respectively.

**Table 1:** Baseline Characteristics of Patients with Stage IA Invasive Mucinous Adenocarcinoma of Shanghai Pulmonary Hospital Cohort

Variables	Surgical procedures		
	Lobectomy (%)	Limited resection (%)	*p*
	(*N* = 316)	(*N* = 87)	
Age			0.156
Mean ± SD	59.1 ± 10.3	60.9 ± 10.3	
≤ 65	229 (72.5)	60 (69.0)	
> 65	87 (27.5)	27 (31.0)	
Sex			0.149
Male	121 (38.3)	26 (29.9)	
Female	195 (61.7)	61 (70.1)	
Smoking			0.626
Non-smoker	258 (81.6)	73 (83.9)	
Smoker	58 (18.4)	14 (16.1)	
Tumor location			0.530
Upper and middle	128 (40.5)	32 (36.8)	
Lower	188 (59.5)	55 (63.2)	
Tumor size, radiological			0.014
≤ 2 cm	199 (63.0)	67 (77.0)	
2.1–3 cm	117 (37.0)	20 (23.0)	
%predicted FEV1			0.103
Mean ± SD	81.4 ± 2.9	80.8 ± 3.4	
CEA			0.432
≤ 5 ng/ml	252 (79.7)	66 (75.9)	
> 5 ng/ml	64 (20.3)	21 (24.1)	
Cardiovascular disease			0.083
Absent	283 (89.6)	72 (82.8)	
Present	33 (10.4)	15 (16.1)	
Diabetes mellitus			0.103
Absent	276 (87.3)	70 (80.5)	
Present	40 (12.7)	17 (19.5)	
VPI			0.141
Absent	263 (83.2)	78 (89.7)	
Present	53 (16.8)	9 (10.3)	
LN status			0.771
Negative	299 (94.6)	83 (95.4)	
Positive	17 (5.4)	4 (4.6)	

FEV1, forced expiratory volume in one second; CEA, carcinoembryonic antigen; VPI, visceral pleural invasion; LN, lymph node

374 (92.8%, 374/403) tumors were pure IMA, and 29 tumors (7.2%, 29/403) were mixed mucinous/nonmucinous ADC: 15 (51.7%,15/29) tumors with acinar pattern, 8 (27.6%, 8/29) tumors with papillary pattern, and 6 (20.7%, 6/29) tumors with acinar and papillary pattern (Supplementary Fig. 1).

#### Outcomes of patients with clinical stage IA invasive lung mucinous adenocarcinomas

**Fig. 1:**
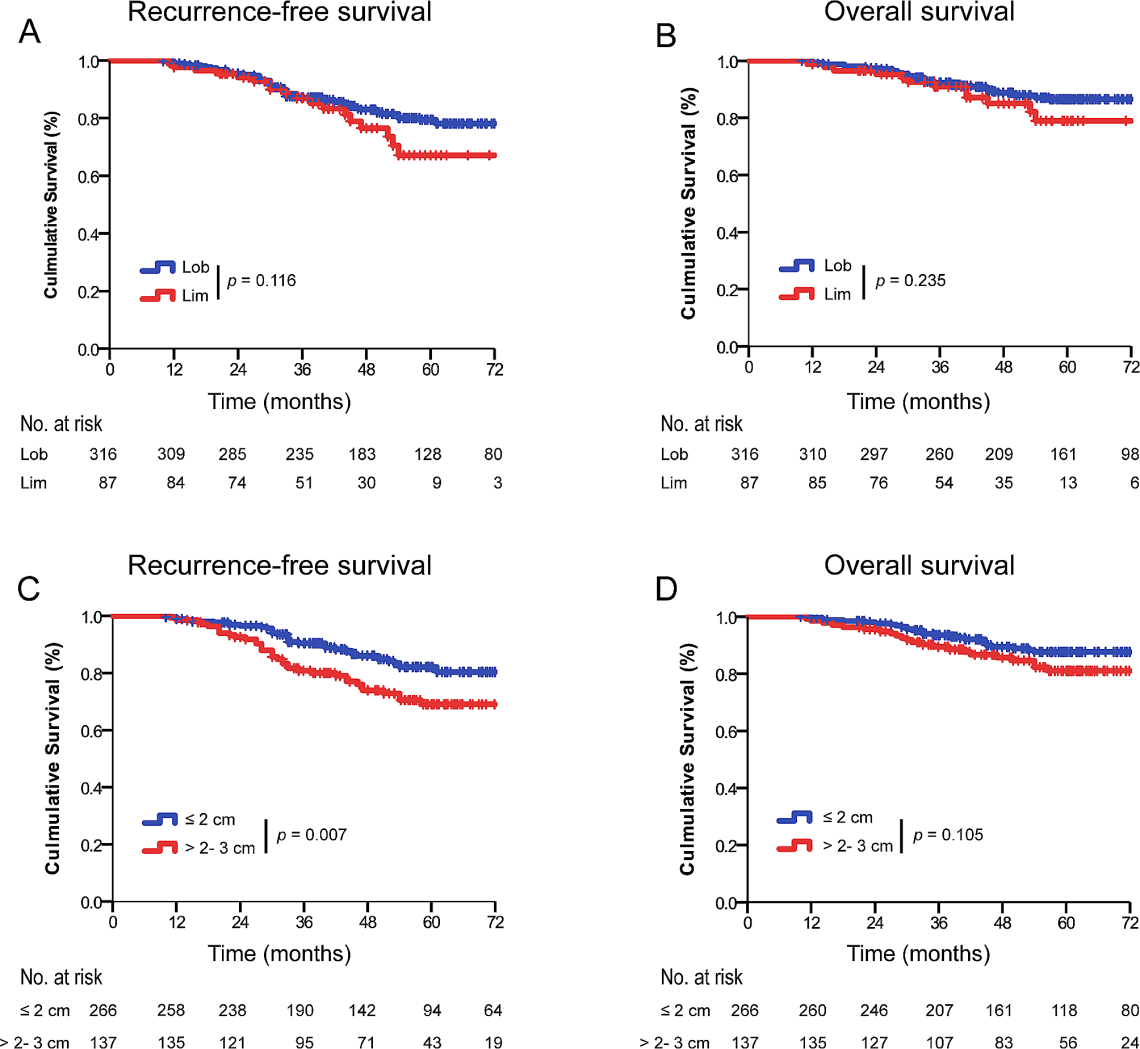
Recurrence-free survival (RFS) and overall survival (OS) in patients with clinical stage IA lung IMA stratified by surgical procedures and tumor size. (**A**) RFS by surgical procedures in patients with clinical stage IA lung IMA. (**B**) OS by surgical procedures in patients with clinical stage IA lung IMA. (**C**) RFS by tumor size for patients with clinical stage IA lung IMA. (**D**) OS by tumor size for patients with clinical stage IA lung IMA Lob, lobectomy; Lim, limited resection

The survival analysis by Log-rank test showed that patients who underwent lobectomy had a similar RFS (5-year RFS: 82.6% *versus*. 79.3%; *p* = 0.116) and OS (5-year OS: 88.3% *versus*. 86.2%; *p* = 0.235) than those who underwent limited resection (Fig. 1A, B). Patients with IMA > 2.1 to 3 cm had worse RFS (5-year RFS: 73.7% *versus.* 86.1%; *p* = 0.007) than those with IMA ≤ 2 cm, whereas similar OS was observed between the two groups (5-year OS: 83.9% *versus*. 89.8%; *p* = 0.105) (Fig. 1C, D).

In further multivariable Cox analysis, lymph node metastasis (hazard ratio [HR], 4.204; 95% confidence interval [CI], 2.126–8.315; *p* < 0.001) and IMA > 2.1 to 3 cm (HR, 1.286; 95% CI, 1.019–1.621; *p* = 0.033) were independent risk factors of worse RFS. In terms of OS, lymph node metastasis (HR, 4.497; 95% CI, 2.106–9.603; *p* < 0.001) was the only independent risk factor (Table 2).

**Table 2:** Cox Proportional-hazards Regression Model for Recurrence-free Survival and Overall Survival in Patients with IMA ≤ 3 cm of Shanghai Pulmonary Hospital Cohort (*n* = 403)

Variables	Recurrence-free survival			Overall survival		
	Univariate	Multivariate		Univariate	Multivariate	
	*p*	HR (95%CI)	*p*	*p*	HR (95%CI)	*p*
Age (> 65 vs. ≤ 65)	0.597			0.663		
Gender (male vs. female)	0.378			0.138		
Smoking (current or ex vs. non-smoker)	0.404			0.756		
Tumor size (2.1–3 cm vs. ≤ 2 cm)	0.008	1.286 (1.019–1.621)	0.033	0.110		
Cardiovascular disease (present vs. absent)	0.397			0.211		
Diabetes mellitus (present vs. absent)	0.149			0.622		
%predicted FEV1 (> 80% vs. ≤ 80%)	0.885			0.267		
CEA (> 5 ng/ml vs. ≤ 5 ng/ml)	0.091	1.344 (0.782–2.309)	0.284	0.246		
VPI (present vs. absent)	0.319			0.622		
Tumor location (upper and middle vs. lower)	0.378			0.870		
Surgical procedure (limited resection vs. lobectomy)	0.170			0.239		
Lymph node status (positive vs. negative)	< 0.001	4.204 (2.126–8.315)	< 0.001	< 0.001	4.497 (2.106–9.603)	< 0.001

HR, harzard ratio; CI, confidence interval; Variables with *P*-value < 0.1 in univariate models were analyzed in multivariate analysis model

FEV1, forced expiratory volume in one second; CEA, carcinoembryonic antigen; VPI, visceral pleural invasion

#### Outcomes of patients with clinical stage IA invasive lung mucinous adenocarcinomas ≤ 2 cm

**Fig. 2:**
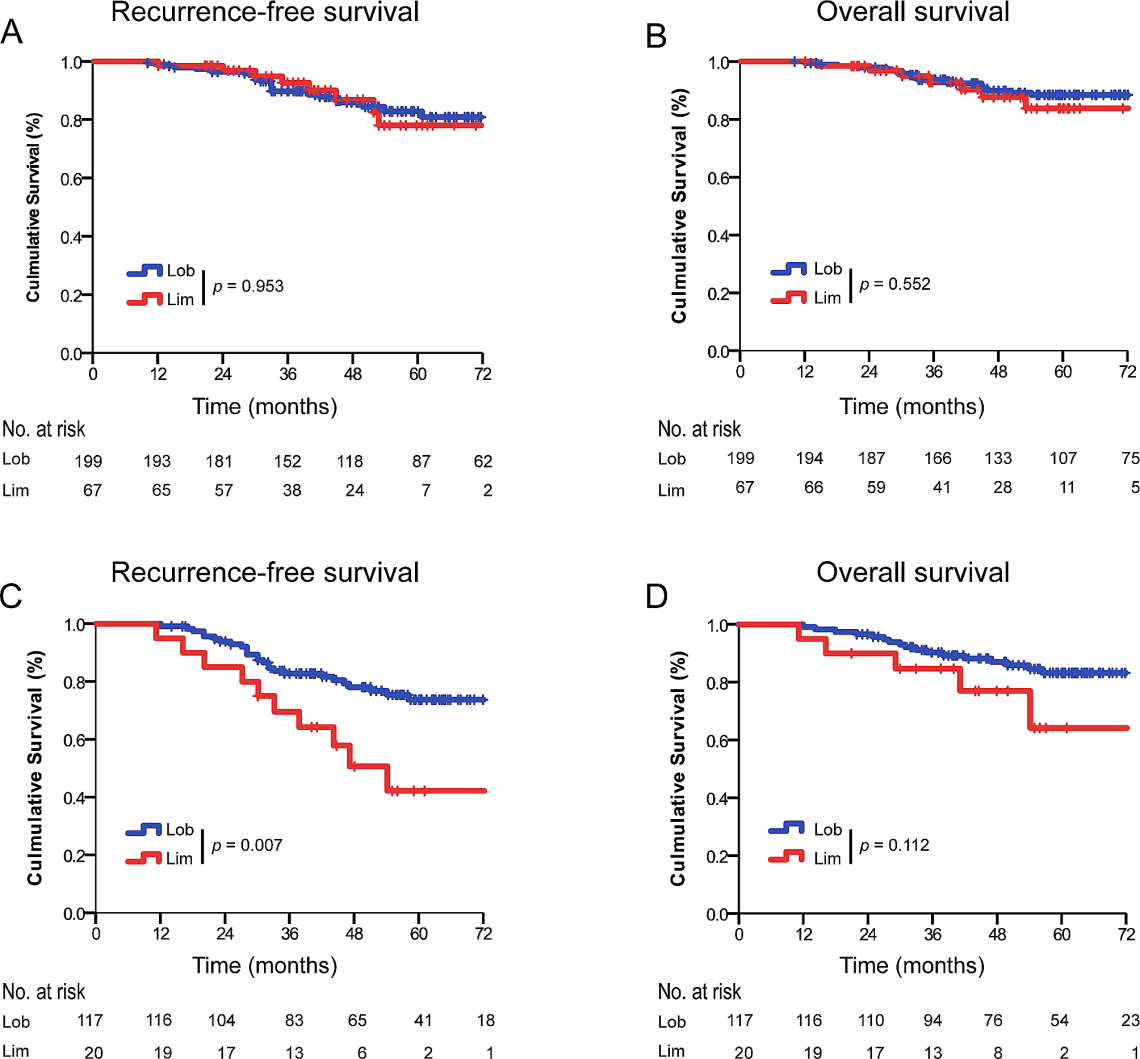
Recurrence-free survival (RFS) and overall survival (OS) in patients with clinical stage IA lung IMA stratified by surgical procedures and tumor size. (**A**) RFS by surgical procedures in patients with clinical stage IA lung IMA ≤ 2 cm. (**B**) OS by surgical procedures in patients with clinical stage IA lung IMA ≤ 2 cm. (**C**) RFS by surgical procedures for patients with clinical stage IA lung IMA > 2 to 3 cm. (**D**) OS by surgical procedures for patients with clinical stage IA lung IMA > 2 to 3 cm Lob, lobectomy; Lim, limited resection

There were 266 patients in the subgroup of IMA ≤ 2 cm, including 67 patients who underwent limited resection and 199 patients who underwent lobectomy. The survival analysis by Log-rank test showed that patients undergoing lobectomy had a similar RFS (5-year RFS: 88.1% *versus.* 85.4%; *p* = 0.953) and OS (5-year OS: 89.9% *versus.* 89.6%; *p* = 0.552) than those who underwent limited resection. (Fig. 2A, B) Multivariable Cox proportional hazards regression model showed that lymph node metastasis was the independent risk factor of worse RFS (HR, 6.981; 95% CI, 2.433–20.034; *p* < 0.001) and OS (HR, 4.296; 95% CI, 1.291–14.297; *p* = 0.020) (Table 3).

**Table 3:** Cox Proportional-hazards Regression Model for Recurrence-free Survival and Overall Survival in Patients with IMA ≤ 2 cm (*n* = 266) and > 2 to 3 cm of Shanghai Pulmonary Hospital Cohort (*n* = 137)

Variables	Recurrence-free survival			Overall survival		
	Univariate	Multivariate		Univariate	Multivariate	
	*p*	HR (95%CI)	*p*	*p*	HR (95%CI)	*p*
IMA ≤ 2 cm						
Age (> 65 vs. ≤ 65)	0.564			0.304		
Gender (male vs. female)	0.181			0.689		
Smoking (current or ex vs. non-smoker)	0.456			0.286		
Cardiovascular disease (present vs. absent)	0.444			0.278		
Diabetes mellitus (present vs. absent)	0.177			0.649		
%predicted FEV1 (> 80% vs. ≤ 80%)	0.475			0.695		
CEA (> 5 ng/ml vs. ≤ 5 ng/ml)	0.141			0.511		
VPI (present vs. absent)	0.468			0.714		
Tumor location (upper and middle vs. lower)	0.465			0.741		
Surgical procedure (limited resection vs. lobectomy)	0.953			0.552		
Lymph node status (positive vs. negative)	< 0.001	6.981 (2.433–20.034)	< 0.001	0.017	4.296 (1.291–14.297)	0.020
IMA > 2.1 to 3 cm						
Age (> 65 vs. ≤ 65)	0.767			0.880		
Gender (male vs. female)	0.648			0.137		
Smoking (current or ex vs. non-smoker)	0.887			0.330		
Cardiovascular disease (present vs. absent)	0.422			0.417		
Diabetes mellitus (present vs. absent)	0.971			0.857		
%predicted FEV1 (> 80% vs. ≤ 80%)	0.906			0.352		
CEA (> 5 ng/ml vs. ≤ 5 ng/ml)	0.263			0.253		
VPI (present vs. absent)	0.845			0.777		
Tumor location (upper and middle vs. lower)	0.664			0.881		
Surgical procedure (limited resection vs. lobectomy)	0.010	2.417 (1.157–5.049)	0.019	0.122		
Lymph node status (positive vs. negative)	0.002	3.444 (1.492–7.949)	0.004	0.005	4.059 (1.495–11.025)	0.005

HR, harzard ratio; CI, confidence interval; Variables with *P*-value < 0.1 in univariate models were analyzed in multivariate analysis model. FEV1, forced expiratory volume in one second; CEA, carcinoembryonic antigen; VPI, visceral pleural invasion

#### Outcomes of patients with clinical stage IA invasive lung mucinous adenocarcinomas 2–3 cm

137 patients were included in the subgroup of IMA > 2.1–3 cm, which 20 patients underwent limited resection and 117 patients underwent lobectomy. There was a significant difference in RFS between lobectomy and limited resection (5-year RFS: 77.8% *versus.* 50.0%; *p* = 0.007) in the group with IMA > 2.1–3 cm, however, similar OS were observed (5-year OS: 85.5% *versus.* 75.0%; *p* = 0.112) (Fig. 2C, D). The multivariable Cox proportional hazards regression model reveals limited resection (HR, 2.417; 95% CI, 1.157–5.049; *p* = 0.019) and lymph node metastasis (HR, 3.444; 95% CI, 1.492–7.949; *p* = 0.004) were the independent risk of worse RFS. Lymph node metastasis (HR, 4.059; 95% CI, 1.495–11.025; *p* = 0.005) was the only independent risk of worse OS (Table 3).

### The SEER cohort

#### Patient characteristics

We identified 480 patients from the SEER database who had stage T1 disease with invasive mucinous adenocarcinoma 403 (403/480 84.0%) or mucinous bronchiolo-alveolar carcinoma 68 (68/480 14.1%) or mixed mucinous and non-mucinous bronchiolo-alveolar carcinoma 9 (9/480 1.9%). Among them, 376 (376/480 78.3%) were underwent lobectomy, and 104 (104/480 21.7%) were treated by limited resection. Clinicopathological characteristics of the patients are summarized in Table 4. There are no significant differences between patients in limited resection group and lobectomy group in terms of sex (*p* = 0.741), age (*p* = 0.116), race/ethnicity (*p* = 0.965), tumor location (*p* = 0.331), and histology (*p* = 0.712). Patients in the limited resection group had smaller tumor size (*p* = 0.002) and fewer lymph nodes involvement (*p* = 0.005) than those in the lobectomy group.

**Table 4:** Baseline Characteristics of Patients with Stage IA Invasive Mucinous Adenocarcinoma of Seer Cohort(*n* = 480)

Variables	Surgical procedures		
	Lobectomy (%)	Limited resection (%)	*p*
	(*N* = 376)	(*N* = 104)	
Age			0.116
Mean ± SD	63.9 ± 10.1	65.6 ± 10.2	
≤ 65	183 (48.7)	51 (49.0)	
> 65	193 (51.3)	53 (51.0)	
Sex			0.741
Male	155 (41.2)	41 (39.4)	
Female	221 (58.8)	63 (60.6)	
Race/ethnicity			0.965
White	303 (80.6)	85 (81.7)	
Black	35 (9.3)	9 (8.7)	
Other	38 (10.1)	10 (9.6)	
Tumor location			0.331
Upper and middle	168 (44.7)	37 (35.6)	
Lower	202 (53.7)	65 (62.5)	
Overlapping	1 (0.3)	0 (0)	
NOS	5 (1.3)	2 (1.9)	
AJCC T stage 7th ed			0.002
T1a	223 (59.3)	79 (76.0)	
T1b	153 (40.7)	25 (24.0)	
Histology			0.712
M-BAC	54 (14.4)	14 (13.5)	
Mix-BAC	8 (2.1)	1 (1.0)	
IMA	314 (83.5)	89 (85.6)	
LN status			0.005
Negative	342 (91.0)	103 (99.0)	
Positive	34 (9.0)	1 (1.0)	

AJCC, American Joint Committee on Cancer; M-BAC, mucinous bronchiolo-alveolar carcinoma; Mix-BAC, mixed mucinous and non-mucinous bronchiolo-alveolar carcinoma; IMA, invasive mucinous adenocarcinoma; LN, lymph node

#### Outcomes of patients with invasive lung mucinous adenocarcinomas ≤ 2 cm

**Fig. 3:**
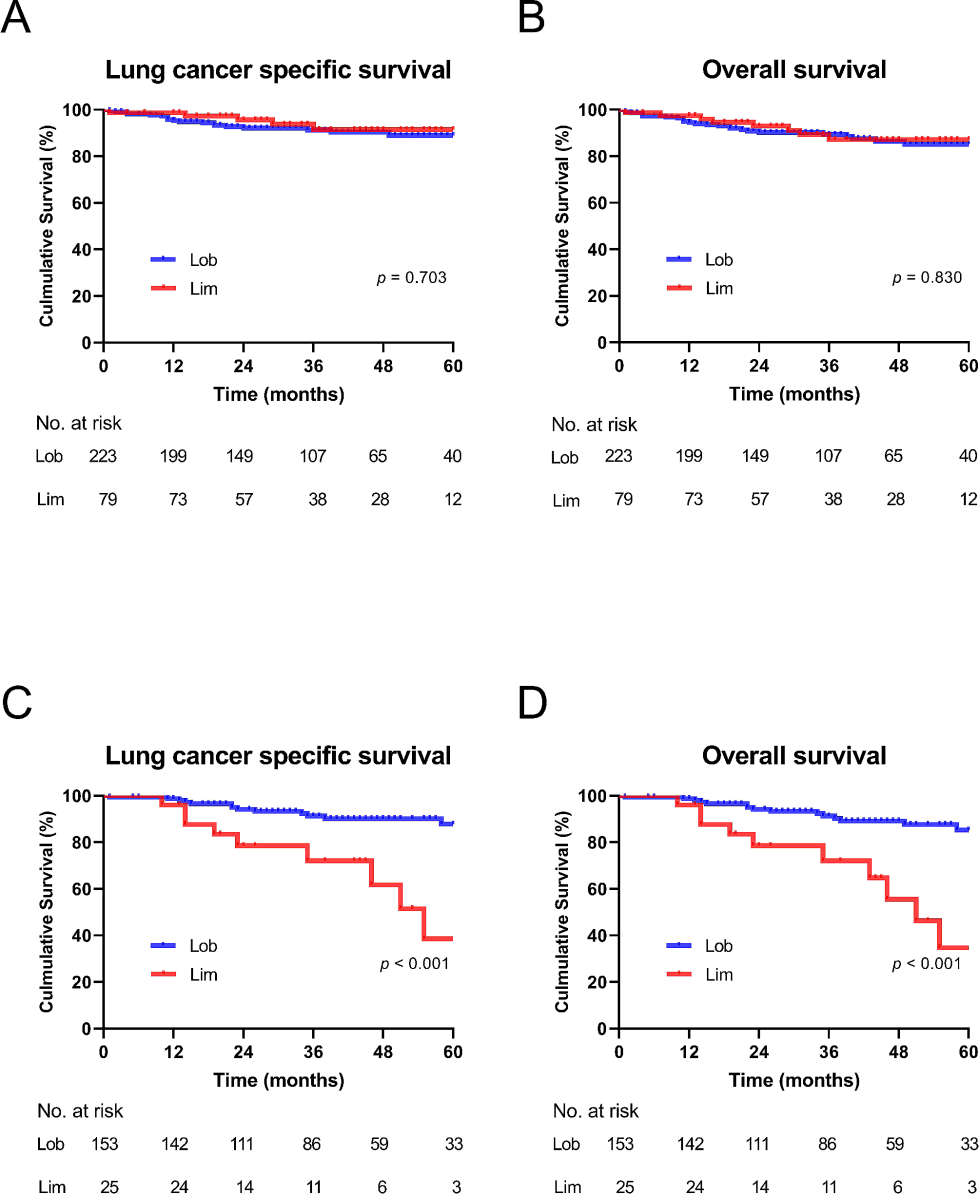
Lung cancer–specific survival (LCSS) and overall survival (OS) in patients with stage T1 lung IMA stratified by surgical procedures and tumor size. (**A**) LCSS by surgical procedures in patients with clinical stage T1 lung IMA ≤ 2 cm. (**B**) OS by surgical procedures in patients with clinical stage T1 lung IMA ≤ 2 cm. (**C**) LCSS by surgical procedures for patients with clinical stage T1 lung IMA > 2 to 3 cm. (**D**) OS by surgical procedures for patients with clinical stage T1 lung IMA > 2 to 3 cm Lob, lobectomy; Lim, limited resection

The survival analysis by log-rank test showed that patients undergoing lobectomy had a similar LCSS (5-year LCSS: 91.0% versus 91.1%; *p* = 0.703) and OS (5-year OS: 88.3% versus 84.8%; *p* = 0.830) than those who underwent limited resection. (Fig. 3A, B) Multivariable Cox proportional hazards regression model showed that lymph node metastasis (HR, 8.234; 95% CI, 3.586–18.904; *p* < 0.001) was the independent risk factor of worse LCSS. And lymph node metastasis (HR, 7.778; 95% CI, 3.730-16.217; *p* < 0.001) was an independent predictive factor of worse OS.

#### Outcomes of patients with invasive lung mucinous adenocarcinomas 2–3 cm

In the group with IMA > 2–3 cm, there was a significant difference in LCSS between lobectomy and limited resection (5-year LCSS: 92.8% versus 64.0%; *p* < 0.001), and similar OS were observed (5-year OS: 90.2% versus 60.0%; *p* < 0.001) (Fig. 3C, D). The multivariable Cox proportional hazards regression model reveals limited resection (HR, 10.066; 95% CI, 3.571–28.376; *p* < 0.001) and lymph node metastasis (HR, 14.346; 95% CI, 4.962–41.476; *p* < 0.001) were the independent risk of worse LCSS. Limited resection (HR, 8.527; 95% CI, 3.355–21.676; *p* < 0.001) and lymph node metastasis (HR, 11.199; 95% CI, 4.188–29.950; *p* < 0.001) were independent risk of worse OS (Table 5).

**Table 5:** Cox Proportional-hazards Regression Model for Recurrence-free Survival and Overall Survival in Patients with IMA ≤ 2 cm (*n* = 302) and > 2 to 3 cm of SEER Cohort (*n* = 178)

Variables	Lung cancer–specific survival			Overall survival		
	Univariate	Multivariate		Univariate	Multivariate	
	*p*	HR (95%CI)	*p*	*p*	HR (95%CI)	*p*
IMA ≤ 2 cm						
Age (> 65 vs. ≤ 65)	0.363			0.758		
Gender (male vs. female)	0.946			0.979		
Race/ethnicity (white vs. non-white)	0.376			0.700		
Tumor location (upper and middle vs. lower)	0.170			0.388		
Surgical procedure (limited resection vs. lobectomy)	0.704			0.830		
Lymph node status (positive vs. negative)	< 0.001	8.234 (3.586–18.904)	< 0.001	< 0.001	7.778 (3.730-16.217)	< 0.001
IMA > 2 to 3 cm						
Age (> 65 vs. ≤ 65)	0.518			0.240		
Gender (male vs. female)	0.391			0.468		
Race/ethnicity (white vs. non-white)	0.634			0.478		
Tumor location (upper and middle vs. lower)	0.552			0.206		
Surgical procedure (limited resection vs. lobectomy)	< 0.001	10.066 (3.571–28.376)	< 0.001	< 0.001	8.527 (3.355–21.676)	< 0.001
Lymph node status (positive vs. negative)	< 0.001	14.346 (4.962–41.476)	< 0.001	< 0.001	11.199 (4.188–29.950)	< 0.001

HR, harzard ratio; CI, confidence interval; Variables with *P*-value < 0.1 in univariate models were analyzed in multivariate analysis model

## Discussion

Based on a special profile of clinical features, gene mutations, radiological presence, clinicopathological characteristics, and clinical outcomes, IMA was regarded as a variant subtype of lung ADC in the 2015 WHO classification [1]. Although several retrospective studies observed the clinical characteristics and pathological features of IMA, the optimal surgical extent for early-stage IMA remains uncertain [4, 5, 18]. In the current study, the Shanghai Pulmonary Hospital cohort, we identified 403 patients with clinical stage IA IMA from the cases of resected lung ADC. We found larger tumor size (> 2 to 3 cm versus ≤ 2 cm) and lymph node metastasis as independent predictors of poor RFS. In the analysis of IMA > 2 to 3 cm subgroup, limited resection was associated with poor RFS compared to lobectomy. However, in the IMA ≤ 2 cm subgroup, limited resection achieved similar prognosis to lobectomy. In the SEER cohort, limited resection and lymph node invoement were both prognostic factors for LCSS and OS in IMA > 2 to 3 cm subgroup. Our findings indicated that patients with clinical stage IA IMA ≤ 2 cm could be candidates for limited resection, whereas lobectomy should be recommended for IMA > 2 to 3 cm to avoid recurrence.

IMA was previously classified as a mucinous bronchiolo-alveolar carcinoma (BAC) in the classification system of lung ADC [2, 19]. Radiographically, BAC present as GGO with/without a central solid region, including (1) a single focus ground-glass nodule, (2) multifocal lesions with the semblable appearance, or (3) dense pneumonic consolidation with mass containing prominent air-bronchograms. IMA exhibit a variety of BAC manifestations [20, 21]. The low invasive nudules usually show a higher proportion of GGO compoment in ADC. In order to minimize surgical trauma and preserve more pulmonary function, limited resection become a great choice for preinvasive and low risk lesions such as ADC *in situ*, minimally invasive ADC and lepidic predominant invasive ADC in early-stage non-small cell lung cancer (NSCLC). Thus, it is natural to assume that tumors with better prognosis could be excised with a smaller resection method.

But on the other hand, the optimal surgical strategy for early-stage NSCLC remains controversial. Although lobectomy with systematic lymph node dissection has been the standard surgical treatment for stage IA NSCLC [22–24], limited resection with great advantage aroused surgeon’s interest. In a previous randomized control trial, patients with peripheral NSCLC ≤ 2 cm that underwent limited resection retained more lung function than those who underwent lobectomy without a compromised prognosis [16]. Another meta-analysis revealed that patients with stage I lung cancer who underwent intentional limited resection achieved comparable survival with lobectomy [23]. In our study, patients with IMA ≤ 2 cm had no differences in prognosis between lobectomy and limited resection. Resently, a milestone randomized control trial, the Japan Clinical Oncology Group (JCOG) 0802 trial [25], shows that patients with tumor size ≤ 2 cm and CTR ≥ 0.5 peripheral NSCLC had a comparable OS and relapse-free survival between segmentectomy and lobectomy in 5 years. They also confirmed that segmentectomy had superiority in preserving pulmonary function (FEV 1.0) at 6 months and 1 year post operation.

Several studies have evaluated the survival difference between limited resection and lobectomy for BAC. Koike et al. [26] performed a prospective study and concluded that limited resection was a potentially curative surgical procedure as an alternative to lobectomy for patients with BAC. Compared to lobectomy, limited resection may offer additional benefits for patients with small size lung cancer in addition to survival. Limited resection allowed for preserving more lung capacity and affording the opportunity for the excision of a second pulmonary neoplasm [27]. Furthermore, limited resection has a significant lower incidence of postoperative complications than lobectomy [28]. Watanabe et al. reported that limited resection could be an appropriate treatment for early-stage BAC [29]. However, few studies have specifically investigated the role of limited resection in mucinous BAC. Our results show that the prognosis was similar between limited resection and lobectomy for patients with IMA ≤ 2 cm. Thus, these findings strongly support that limited resection is an alternative procedure for patients with IMA ≤ 2 cm.

Recently, limited studies have discussed about using imaging features to identify early-stage IMA [30]. Unfortunately, applying these imaging features to guide surgical management is still difficult due to their inaccuracy. Intraoperative frozen section diagnosis could be an effective method to guide surgical strategy for early-stage invasive lung ADC [31]. Walts et al. [32] found that the accuracy of frozen section diagnosis for invasive lung ADC was greater than 97%. Liu et al. [31] used frozen section diagnosis to guide limited resection for peripheral early-stage lung ADC and found a concordance rate of 96.6% between frozen section diagnosis and final pathological diagnosis. Thus, they concluded that intraoperative frozen section diagnosis could be a method to guide resection strategy for early-stage ADC. Furthermore, intraoperative frozen section can aid in surgical decisions. According to our research, when IMA ≤ 2 cm in preoperative CT discovered intraoperatively by frozen section, limited resection is permissible as long as a 2 cm resection margin ensured. If the IMA 2–3 cm cm in preoperative CT diagnosis intraoperatively by frozen section, a complementary lobectomy should be performed.

Lymph nodes involvement is an crucial prognostic factor in early-stage lung cancer [33, 34]. Regardless of whether the IMA ≤ 2 cm or 2–3 cm, the status of the lymph nodes remains the most significant factor influencing prognosis in our study. A cautious and comprehensive preoperative diagnosis and staging of lymph nodes will significantly benefit the prognosis of early-stage IMA. When the patient’s lymph node is suspected to be positive in preoperative CT, further examinations such as positron emission tomography (PET) and endobronchial ultrasound (EBUS) will be performed. It is a rare occurrence to have unexpected macroscopic nodal involvement discovered intraoperatively after negative CT/PET-CT and negative EBUS [35]. Even if unsuspected N2 discovered intraoperatively, a comprehensive adjuvant therapy should be administered for a more favorable the postoperative prognosis [36].

We acknowledge that there were some limitations in our study. First, there were insufficient cases that underwent limited resection to allow us for further analysis of the risk factors of prognosis between segmentectomy and wedge resection. Due to the retrospective nature of this study, the patients selection bias biases were unavoidable. Future prospective studies with less patient selection bias will be conducted to further validation of the impact of surgical methods on the prognosis of IMA. Second, The relative short observation period may reduce the power to analyze the difference in prognosis. Finally, it’s difficult to diagnose a certain histopathological subtype of lung tumor based on preoperative CT alone in clinical practice. Use of frozen section for the diagnosis of IMA may remedy this limitation in future studies.

## Conclusion

In conclusion, patients with clinical stage IA IMA ≤ 2 cm could be candidates for limited resection, whereas limited resection should be performed cautiously in IMA > 2 to 3 cm. Future studies with larger sample size are needed to verify the appropriate surgical extent for IMA.

### Electronic supplementary material

Below is the link to the electronic supplementary material.

Supplementary Figure 1: Distribution of Histologic Subtypes of Invasive Mucinous Adenocarcinomas

## Data Availability

The datasets used and analyzed during the current study are available from the corresponding author on reasonable request.

## Abbreviations

WHO: World Health Organization
ADC: Adenocarcinoma
IMA: Invasive mucinous adenocarcinom
GGO: Ground-glass opacity
SEER: Surveillance, Epidemiology, and End Results
CT: Computed tomography
VPI: Visceral pleural invasion
RFS: Recurrence-free survival
LCSS: Lung cancer–specific survival
OS: Overall survival
CEA: Carcinoembryonic antigen
BAC: Bronchiolo-alveolar carcinoma
NSCLC: Non-small cell lung cancer
PET: Positron emission tomography
EBUS: Endobronchial ultrasound

## References

[1] Travis WD, Brambilla E, Nicholson AG (2015). The 2015 World Health Organization Classification of Lung Tumors: impact of genetic, clinical and radiologic advances since the 2004 classification. J Thorac Oncol.

[2] Travis WD, Brambilla E, Noguchi M (2011). International Association for the Study of Lung Cancer/American Thoracic Society/European Respiratory Society International Multidisciplinary Classification of Lung Adenocarcinoma. J Thorac Oncol.

[3] Hwang DH, Sholl LM, Rojas-Rudilla V (2016). KRAS and NKX2-1 mutations in Invasive Mucinous Adenocarcinoma of the lung. J Thorac Oncol.

[4] Lee HY, Cha MJ, Lee KS (2016). Prognosis in Resected Invasive Mucinous adenocarcinomas of the lung: related factors and comparison with Resected Nonmucinous Adenocarcinomas. J Thorac Oncol.

[5] Luo J, Wang R, Han B (2016). Analysis of the clinicopathologic characteristics and prognostic of stage I invasive mucinous adenocarcinoma. J Cancer Res Clin Oncol.

[6] Wisnivesky JP, Henschke CI, Swanson S (2010). Limited resection for the treatment of patients with stage IA lung cancer. Ann Surg.

[7] Zhong C, Fang W, Mao T (2012). Comparison of thoracoscopic segmentectomy and thoracoscopic lobectomy for small-sized stage IA lung cancer. Ann Thorac Surg.

[8] Blasberg JD, Pass HI, Donington JS (2010). Sublobar resection: a movement from the Lung Cancer Study Group. J Thorac Oncol.

[9] Yoshida Y, Manaka T, Nitadori JI (2019). A comparison between 2- and 3-dimensional approaches to solid component measurement as radiological criteria for sublobar resection in lung adenocarcinoma = 2 cm in size</at. Surg Today.

[10] Chiang XH, Hsu HH, Hsieh MS (2019). Propensity-matched analysis comparing Survival after Sublobar Resection and Lobectomy for cT1N0 Lung Adenocarcinoma. Ann Surg Oncol.

[11] Goldstraw P, Chansky K, Crowley J (2016). The IASLC Lung Cancer Staging Project: proposals for revision of the TNM Stage groupings in the Forthcoming (Eighth) Edition of the TNM classification for Lung Cancer. J Thorac Oncol.

[12] Kocaturk CI, Gunluoglu MZ, Cansever L (2011). Survival and prognostic factors in surgically resected synchronous multiple primary lung cancers. Eur J Cardiothorac Surg.

[13] Sihoe AD, Van Schil P (2014). Non-small cell lung cancer: when to offer sublobar resection. Lung Cancer.

[14] Koike T, Yamato Y, Yoshiya K (2003). Intentional limited pulmonary resection for peripheral T1 N0 M0 small-sized lung cancer. J Thorac Cardiovasc Surg.

[15] Koike T, Koike T, Yoshiya K (2013). Risk factor analysis of locoregional recurrence after sublobar resection in patients with clinical stage IA non-small cell lung cancer. J Thorac Cardiovasc Surg.

[16] Koike T, Koike T, Sato S (2016). Lobectomy and limited resection in small-sized peripheral non-small cell lung cancer. J Thorac Dis.

[17] Tsutani Y, Miyata Y, Nakayama H (2014). Sublobar resection for lung adenocarcinoma meeting node-negative criteria on preoperative imaging. Ann Thorac Surg.

[18] Travis WD, Asamura H, Bankier AA (2016). The IASLC Lung Cancer Staging Project: proposals for Coding T Categories for Subsolid Nodules and Assessment of Tumor Size in Part-Solid tumors in the Forthcoming Eighth Edition of the TNM classification of Lung Cancer. J Thorac Oncol.

[19] Shim HS, Kenudson M, Zheng Z (2015). Unique Genetic and Survival Characteristics of Invasive Mucinous Adenocarcinoma of the lung. J Thorac Oncol.

[20] Gandara DR, Aberle D, Lau D (2006). Radiographic imaging of bronchioloalveolar carcinoma: screening, patterns of presentation and response assessment. J Thorac Oncol.

[21] Austin JH, Garg K, Aberle D (2013). Radiologic implications of the 2011 classification of adenocarcinoma of the lung. Radiology.

[22] Howington JA, Blum MG, Chang AC (2013). Treatment of stage I and II non-small cell lung cancer: diagnosis and management of lung cancer, 3rd ed: American College of Chest Physicians evidence-based clinical practice guidelines. Chest.

[23] Cao C, Chandrakumar D, Gupta S (2015). Could less be more?-A systematic review and meta-analysis of sublobar resections versus lobectomy for non-small cell lung cancer according to patient selection. Lung Cancer.

[24] Cao C, Manganas C, Ang SC, Yan TD (2012). A meta-analysis of unmatched and matched patients comparing video-assisted thoracoscopic lobectomy and conventional open lobectomy. Ann Cardiothorac Surg.

[25] Shimoyama R, Tsutani Y, Wakabayashi M (2020). A multi-institutional randomized phase III trial comparing anatomical segmentectomy and wedge resection for clinical stage IA non-small cell lung cancer in high-risk operable patients: Japan Clinical Oncology Group Study JCOG1909 (ANSWER study). Jpn J Clin Oncol.

[26] Koike T, Togashi K, Shirato T (2009). Limited resection for noninvasive bronchioloalveolar carcinoma diagnosed by intraoperative pathologic examination. Ann Thorac Surg.

[27] Ginsberg RJ, Rubinstein LV (1995). Randomized trial of lobectomy versus limited resection for T1 N0 non-small cell lung cancer. Lung Cancer Study Group. Ann Thorac Surg.

[28] Ichinose J, Yamamoto H, Aokage K et al. Real-world perioperative outcomes of segmentectomy versus lobectomy for early-stage lung cancer: a propensity score-matched analysis. Eur J Cardiothorac Surg 2022; 63. 10.1093/ejcts/ezac529 36321968

[29] Watanabe S, Watanabe T, Arai K (2002). Results of wedge resection for focal bronchioloalveolar carcinoma showing pure ground-glass attenuation on computed tomography. Ann Thorac Surg.

[30] Raz DJ, Kim JY, Jablons DM (2007). Diagnosis and treatment of bronchioloalveolar carcinoma. Curr Opin Pulm Med.

[31] Liu S, Wang R, Zhang Y (2016). Precise diagnosis of Intraoperative Frozen section is an effective method to Guide Resection Strategy for Peripheral Small-Sized Lung Adenocarcinoma. J Clin Oncol.

[32] Walts AE, Marchevsky AM (2012). Root cause analysis of problems in the frozen section diagnosis of in situ, minimally invasive, and invasive adenocarcinoma of the lung. Arch Pathol Lab Med.

[33] Dezube AR, Mazzola E, Bravo-Iñiguez CE (2021). Analysis of Lymph Node Sampling Minimums in Early Stage Non-small-cell Lung Cancer. Semin Thorac Cardiovasc Surg.

[34] Darling GE (2020). Lymph node assessment in early stage non-small cell lung cancer lymph node dissection or sampling?. Gen Thorac Cardiovasc Surg.

[35] Yang CJ, D’Amico TA, Berry MF (2016). Frozen section of N2 nodes is invaluable whenever unexpected suspicious operative findings are encountered. J Thorac Cardiovasc Surg.

[36] Yang CF, Kumar A, Gulack BC (2016). Long-term outcomes after lobectomy for non-small cell lung cancer when unsuspected pN2 disease is found: a National Cancer Data Base analysis. J Thorac Cardiovasc Surg.

