# Supplementary figures and images for: Limited resection is comparable to lobectomy for tumor size ≤ 2 cm pulmonary invasive mucinous adenocarcinoma
Source: World J Surg Oncol. 2024 Apr 25;22:109. doi: 10.1186/s12957-024-03387-5 (PMC11044566; doi:10.1186/s12957-024-03387-5)

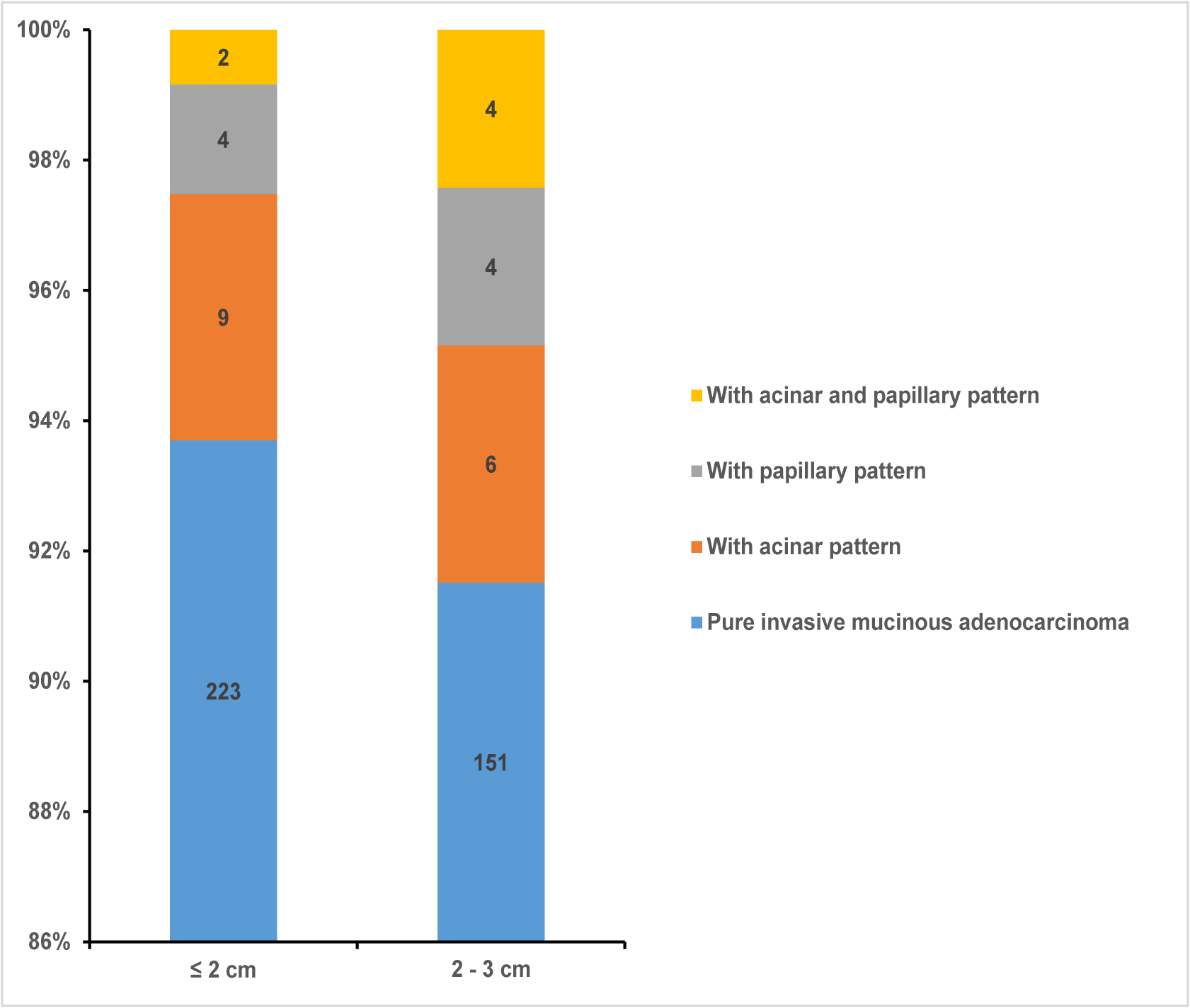

Supplement: Supplementary file 1 — Supplementary Figure 1: Distribution of Histologic Subtypes of Invasive Mucinous Adenocarcinomas [file 12957_2024_3387_MOESM1_ESM.png]
